# Reconstruction of the human lower esophageal sphincter based on ultra-mill imaging for biomechanical analysis

**DOI:** 10.3389/fphys.2023.1128903

**Published:** 2023-07-20

**Authors:** Jack Xu, Savindi Wijenayaka, Recep Avci, Leo K. Cheng, Peng Du

**Affiliations:** Auckland Bioengineering Institute, University of Auckland, Auckland, New Zealand

**Keywords:** gastroesophageal junction, GEJ, FEM, visible human model, multi-scale modeling, biomechanics modeling

## Abstract

**Introduction:** The lower esophageal sphincter (LES) controls the passage into the stomach and prevents reflex of contents into the esophagus. Dysfunctions of this region typically involves impairment of muscular function, leading to diseases including gastro-esophageal reflux disease and achalasia. The main objective of this study was to develop a finite element model from a unique human LES dataset reconstructed from an ultra-mill imaging setup, and then to investigate the effect of anatomical characteristics on intraluminal pressures.

**Methods:** A pipeline was developed to generate a mesh from a set of input images, which were extracted from a unique ultra-mill sectioned human LES. A total of 216 nodal points with cubic Hermite basis function was allocated to reconstruct the LES, including the longitudinal and circumferential muscles. The resultant LES mesh was used in biomechanical simulations, utilizing a previously developed LES mathematical model based on the Visible Human data to calculate intraluminal pressures. Anatomical and functional comparisons were made between the Ultra-mill and Visible human models.

**Results:** Overall, the Ultra-mill model contained lower cavity (1,796 vs. 5,400 mm^3^) and muscle (1,548 vs. 15,700 mm^3^) volumes than the Visible Human model. The Ultra-mill model also developed a higher basal pressure (13.8 vs. 14.7 mmHg) and magnitude of pressure (19.8 vs. 18.9 mmHg) during contraction. Out of all the geometric transformations (i.e., uniform enlargement of volume, lengthening along the center-axis, dilation of the diameter, and increasing muscle thickness), the muscle volume was found to be the main contributor of basal and magnitude of pressures. Increases in length also caused proportional increases to pressures, while dilation of diameter had a less influential reverse effect.

**Discussion:** The findings provide information on interindividual variability in LES pressure and demonstrates that anatomy has a large influence on pressures. This model forms the basis of more complex simulations involving food bolus transport and predicting LES dysfunctions.

## 1 Introduction

The human esophagus is a 25–30 cm long muscular organ connecting the pharynx to the stomach, and it allows passage of ingested food boluses through the lower esophageal sphincter (LES) (Mahadevan, 2020). Swallowing initiates “primary” peristaltic waves that begin orally and travel towards the LES. If the initial primary waveform is not enough to clear the bolus from the esophagus, then a “secondary” is initiated, prolonging peristalsis until the bolus is moved through the LES. Both primary and secondary waveforms are centrally mediated (Goyal and Chaudhury, 2008). The human esophagus transitions from straited muscles in the cervical region to smooth muscles in the thoracic region. The LES is comprised of tonic muscle that is different from the main esophageal body, and innervated by both inhibitory and excitatory neurons, forming a complex set of coordinated movements during swallowing (Goyal and Chaudhury, 2008). Clinical disorders of esophageal motility, such as achalasia and reflux, can be classified on the basis of disorders of the inhibitory and excitatory innervations and the smooth muscles (Paoletti et al., 2021; Savarino et al., 2022).

A number of techniques have been applied clinically to diagnose LES disorders. In addition to imaging modalities such as endoscopy, ultrasound, electrical impedance and video-fluoroscopy (Furlow, 2004; Krugmann et al., 2013; Wu et al., 2020), functional recordings such as pH monitoring have been applied to monitor acid reflux in the LES over an extended period (24 h) (Mitchell et al., 2001). Pressure recordings such as high-resolution manometry involving water-perfused or solid-state pressure sensors provide detailed pressure profiles of esophageal contractions (Nayar et al., 2005; Fox and Bredenoord, 2008). Alternatively, multichannel intraluminal impedance has also been applied to measure the change in the impedance during swallowing, by alternating currents between pairs of electrodes on a specialized catheter in contact with the esophageal lumen (Tutuian et al., 2003). An advantage of measuring impedance over multiple locations is that it allows for the detection of bolus movement in both directions, irrespective of pH, making it capable of detecting both acid and non-acid refluxes.

In addition to measurement methods, advances in computation and visualization techniques have been critical to the effective processing and interpretation of various measurements. Finite element method (FEM) based mathematical models of the LES have been developed based on realistic human anatomy to simulate intraluminal pressure development and/or fluid dynamics (Yassi et al., 2009; Du et al., 2016; Acharya et al., 2021). Nicosia and Brasseur proposed a model based on distinctive passive and active components of circular muscle tension within the upper esophageal sphincter during bolus transport (Nicosia and Brasseur, 2002). The authors utilized video-fluoroscopy data and approximated the esophagus as a 3D-cylinder, and simulated the deformation based on the recorded intraluminal pressures. Other modeling studies have investigated the biomechanics behind the “buckling” of mucosal fold in the esophagus and suggested its importance in maintaining the normal esophageal function (Liao et al., 2007). A previous study utilized Visible Human male data to reconstruct the LES for biomechanical analysis (Yassi et al., 2009). The model predicted that the LES produced a resting pressure of 13.43 mmHg and a peak pressure of 33.30 mmHg. The inclusion of crural contribution, said to be 56% of the pressure contribution in literature, led to higher pressures of 25.8 and 61.24 mmHg, a closely matching 53% pressure contribution (Yassi et al., 2009).

The main objective of this study was to create an anatomically realistic model of the human LES from imaging data. A secondary objective was to compare the intraluminal pressure developed of the new model to the existing LES model (Yassi et al., 2009). The results of this study would determine the impact of LES anatomy on functions and improve the reliability of the simulation results.

## 2 Methods

**FIGURE 1:**
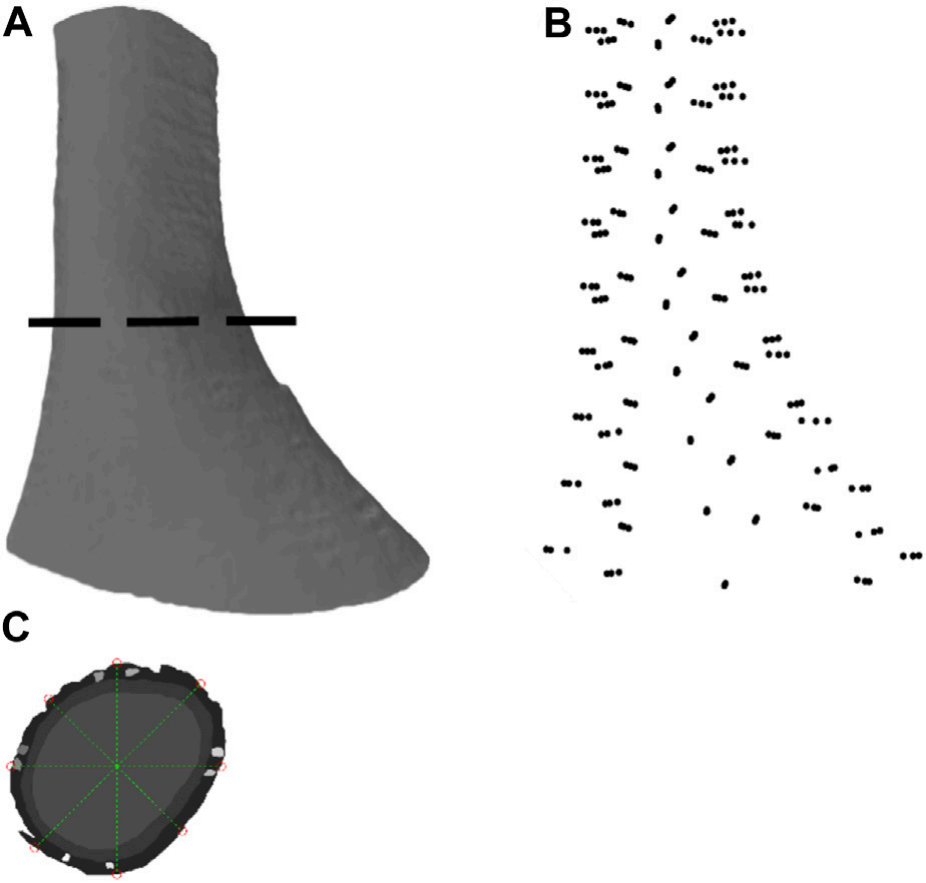
Image segmentation and anatomical model development based on an ultra-mill image dataset of human LES. **(A)** Lateral view of the human LES region, generated through ball pivoting algorithm surface reconstruction performed on the data cloud. **(B)** A data cloud of points obtained from node selection. **(C)** Demarcation of the nodes from the selected points around a segmented section.

Existing high-resolution images of the human LES were used to reconstruct the anatomical model (Yassi et al., 2010). The LES specimen was taken from a cadaver, and imaged using an ultra-mill setup with the cross-section of the sample imaged sequentially by milling at a depth of 50 µm and staining the top surface with May Grunwald solution (Gerneke et al., 2007; Yassi et al., 2010). The images were taken at 8.2 µm/pixel resolution by an 8-megapixel camera, and the resulting images were 7000 × 5816 pixels and covered a field of view of 58 cm × 48 cm. The images were later segmented to distinguish between the longitudinal and circumferential muscle layer arrangements in the LES (Zifan et al., 2017) (Figure 1A). The LES was reconstructed from the ultra-mill dataset (i.e., the Ultra-mill model) and compared against an existing benchmark model (i.e., the Visible Human model) (Yassi et al., 2009).

### 2.1 Anatomical model development

The segmented ultra-mill images were further processed using MATLAB (2021a, MathWorks, Natick, MA, United States). In order to allow a fair comparison with the Visible Human model (Yassi et al., 2009), the segmented ultra-mill images were grouped as LM and CM layers (Figure 1C). A 2D logical array was created from each merged image sequentially by tracing the outline of the tissues and then filling in any remaining holes in the image. For each image, the centroid of the tissue was obtained, and then 8 straight lines originating from the centroid were drawn at equidistant angles from the horizontal line. The intercepts of these lines with the wall boundary were taken as data points for the mesh (Figure 1B). In order to be consistent with the Visible Human FEM mesh setup (Yassi et al., 2009), nine slices at equal distances in the *z*-direction were sampled, from which a total of 216 node points were obtained (24 points per sampled image slice; 8 from serosal surface, 8 from LM/CM interface, 8 from the mucosal surface), with derivatives updated based on the data cloud (Oberhofer et al., 2019).

**FIGURE 2:**
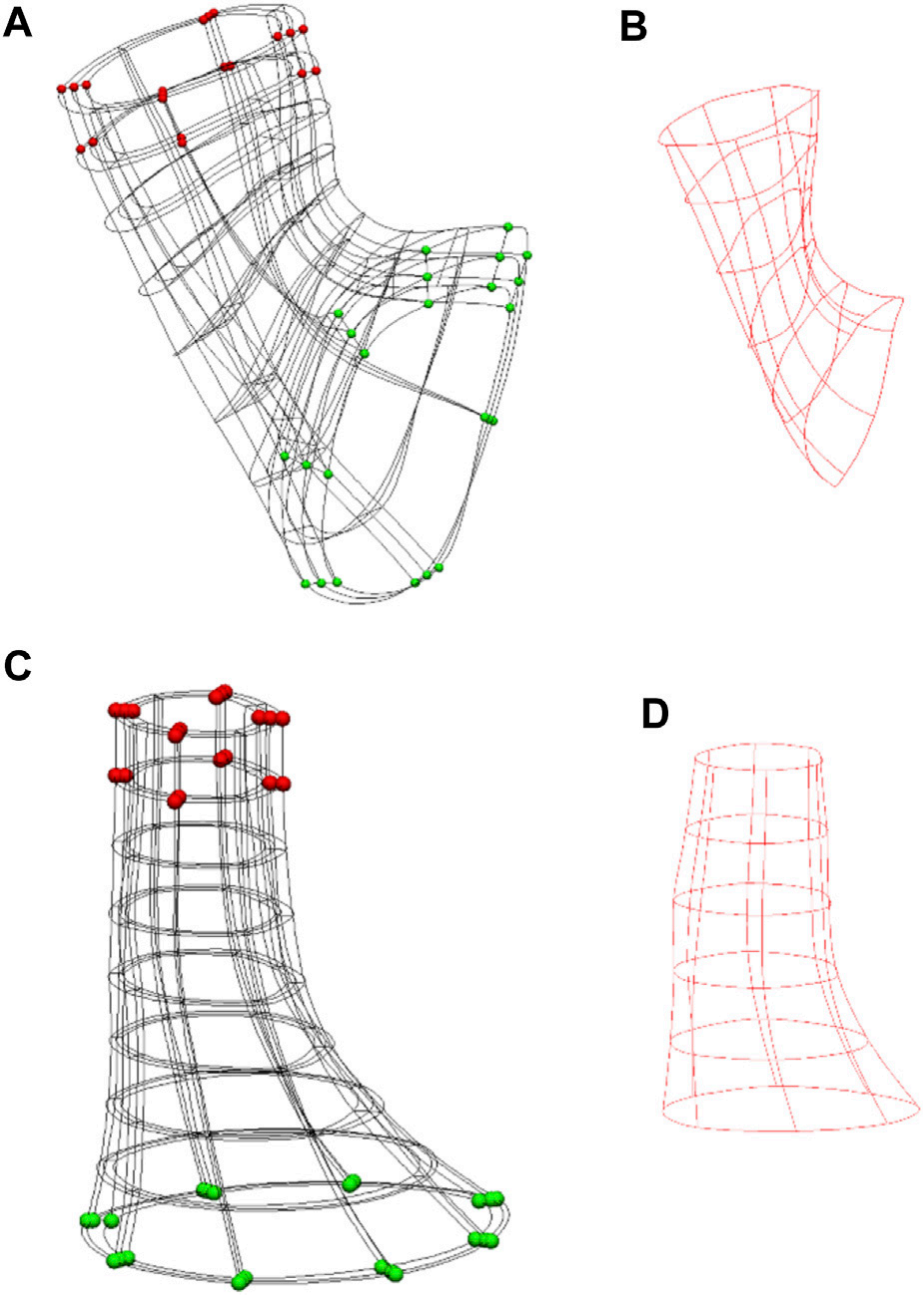
Anatomical models of the Ultra-mill and Visible Human meshes, and their simulation setups. **(A)** The setup of the previously developed Visible Human model (Yassi et al., 2009). **(B)** The Visible Human model cavity mesh. **(C)** The equivalent LES model reconstructed from the Ultra-mill dataset. **(D)** The Ultra-mill data cavity mesh. Boundary conditions are visualized as spheres. Green spheres are fixed in all directions. Red spheres are a grouping of nodes fixed in either the x-z or *y*-*z* directions. Applied boundary conditions help to reduce torsion, while allowing contraction.

Cubic Hermite basis functions were used to ensure derivative continuity between the elements. The outcome was a smoothed 3D mesh with derivatives and nodes arranged at 45° intervals from the in-plane centroid of the LES lumen (Figure 2C). As the muscularis layers consist of an outer LM layer followed by an inner CM layer, the fiber directions were separately assigned for both layers.

A rigid intra-luminal cavity mesh was introduced to simulate the pressure change in the LES model, as previously described (Yassi et al., 2009). The elements were organized as wedge shaped elements with cubic Hermite basis functions forming an intraluminal cavity mesh, as shown in Figures 2B, D. In the present study, the cavity mesh was formed from the same nodes as the inner layer of the LES mesh, with the addition of a centerline of nodes. The centerline was created from the centroids of all image slices except the most proximal layer and the two most distal layers.

### 2.2 Simulation setup

The Guccione transversely isotropic constitutive relation was applied to model the mechanical properties of the muscularis layers of the LES (Guccione et al., 1991). The constitutive law models the mechanical behavior of a tissue in 3D and also includes the resistance to shear. The strain-energy density function of this constitutive relation takes an exponential form as follows,

$$W=\frac{C}{2}\left(e^{Q}-1\right)$$ (2.1)

where,

$$Q=2C_{1}\left(E_{11}+E_{22}+E_{33}\right)+C_{2}E_{11}^{2}+C_{3}\left(E_{22}^{2}+E_{33}^{2}+2E_{23}^{2}\right)+2C_{4}\left(E_{12}^{2}+E_{13}^{2}\right)$$ (2.2)

The properties of the LM and CM layers were described by five material parameters (i.e., C, C_1_-C_4_), the values of which (i.e., 1, 5, 195, 185, and 0.1, respectively) were based on previously reported values (Yassi et al., 2009), from which a perturbation analysis was performed by increasing the value of each parameter independently by 100% in 25% increments (Appendix 1).

Contractions of the LES muscles were modeled by adding an additional term to the passive stress tensor in the following equation,

$$T^{MN}=\frac{1}{2}\left(\frac{\partial \bar{W}}{\partial E_{MN}}+\frac{\partial \bar{W}}{\partial E_{NM}}\right)-pC^{MN}+TJ\frac{\partial X_{M}}{\partial x_{1}}\frac{\partial X_{N}}{\partial x_{1}}$$ (2.3)

where *J* is the determinant of deformation tensor, *W* is the stain energy function, and *p* is the hydrostatics pressure term. The M and N terms are the normal of the surface and directions of the forces respectively. The extra terms altered the T component of the Cauchy stress tensor to include the active tension generated by the fiber (Hunter et al., 1998), which was assumed to occur in the direction of the main axis of the fibers and that the transverse and shear strains had no effect on the active tension generated by the muscles. T denotes a calcium-dependent Hill equation as follows,

$$T\left(\lambda ,Ca_{actn}\right)==\frac{{\left(Ca_{actn}\cdot {\left[{Ca}^{2+}\right]}_{\max}\right)}^{h}}{{\left(Ca_{actn}\cdot {\left[Ca^{2+}\right]}_{\max}\right)}^{h}+{\left(c_{50}\right)}^{h}}T_{ref}\left[1+\beta \left(\lambda -1\right)\right]$$ (2.4)

where Ca_actn_ is a dimensionless parameter in the range of 0–1 representing the activation level, Ca_max_ is the calcium concentration required for maximum activation, and c_50_ is the intracellular concentration that gives 50% of the maximum activated tension. In addition to the calcium concentrations, the model depends on its muscle fiber extension ratio *λ*. The muscle fiber extension ratio is calculated by dividing the current length of the fiber by the resting length. T_ref_ is the tension developed when *λ* is one and at maximum Ca_actn_. β is a constant with no units, and h is the coefficient of the Hill equation. The previously reported parameter values were adopted for this investigation (T_ref_ = 100 kPa, *β* = 1.45, h = 3, Ca_max_ 1 mM) (Yassi et al., 2009).

To prevent the translation of the intraluminal cavity mesh, the bottom nodes were fixed in all directions. This step ensured that the contraction of the esophagus would cause the region to travel orally, as reported in the literature (Winans, 1972). Furthermore, to prevent significant torsional effects from occurring, the nodes highlighted in red were fixed in either the x-z or *y*-*z* directions as shown in Figures 2A, C. The shared intraluminal nodes between the cavity and LES meshes were coupled and free to move in all directions. The material property of the cavity mesh was set to rigid, such that any stress applied to its surface would directly translate to a change in pressure. An incompressible mesh would mean that the pressures applied to the cavity elements would not cause any deformation of the elements themselves and would directly translate to an increase in pressure. Ordinarily, an incompressible inner mesh would prevent the model from contracting altogether. To counteract this, one of the centerline nodes was designated the “valve node” and was given free movement in the *z*-direction, as shown in Figures 2C, D. This setup allows for limited deformation of the GEJ mesh while allowing the monitoring of pressures. The remaining centerline nodes were fixed to help maintain the shape of the LES.

Simulations of the GEJ model were carried out using a single Intel Xeon Gold 6524 3.1 GHz 18C/36T with 2 TB memory. Numerical simulations were all carried out using the CMISS software package. The Newton-Raphson method was used to acquire the solution iteratively, and a full update of the solution was performed at each iteration of the method. Numerical convergence of the setup and mesh was provided in a previous study (Yassi et al., 2009).

Three simulations were performed to compare the intraluminal pressure profile between the Ultra-mill model and the Visible Human model over a full activation cycle (i.e., Ca_actn_ from 0 to 1 in 0.04 increments). The basal pressure was taken at Ca_actn_ = 0.45, in keeping with the assumption that the basal pressure is usually taken at 45% of the time taken to reach pressure during manometric recordings (Yassi et al., 2009). To further explore the impact of anatomy on the intraluminal pressure development, four perturbation studies on the Ultra-mill model were performed: 1) uniform enlargement of volume, 2) lengthening along the center-axis, 3) dilation of the diameter, 4) increasing muscle thickness, all of which were performed by applying a constant scaling factor from 1 to 2 in 0.25 increments.

## 3 Results

### 3.1 Anatomical comparisons between the ultra-mill and visible human models

**FIGURE 3:**
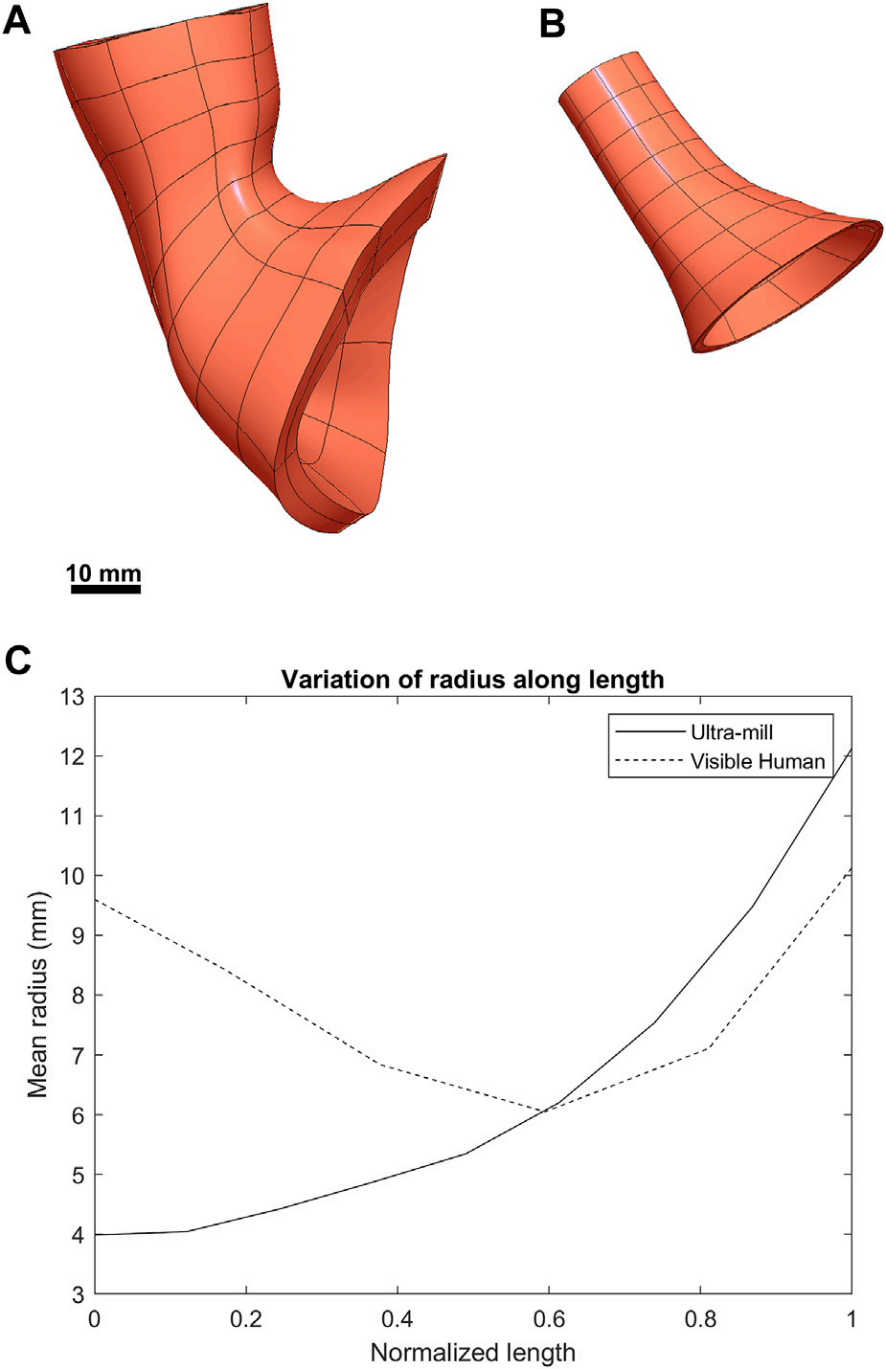
Visual comparison between the two anatomies. **(A)** The previously developed Visible Human LES mesh (Yassi et al., 2009). **(B)** The LES mesh made from the ultra-mill dataset. **(C)** Comparison of the average radius of both meshes along their normalized lengths.

The anatomical differences between the Ultra-mill and Visible Human LES models are illustrated in Figures 3A, B. While the general shape of the Ultra-mill model is roughly straight, the Visible Human model has an obvious bend. The distal end of both models, where the LES transitions into the cardia of the stomach, was dilated compared to the midsection of the Visible Human model (200%) and Ultra-mill model (170%). The Ultra-mill model was also smaller than the Visible Human model, possessing a smaller average width (6 ± 2 vs. 8 ± 2 mm), shorter centerline length (24.5 vs. 48.3 mm), and smaller cavity volume (1,796 vs. 5,400 m^3^). In addition, the Visible Human model has noticeably thicker LM and CM walls, which resulted in a comparatively higher muscle volume (15,700 vs. 1,548 mm^3^), as shown in Table 1.

**TABLE 1:** Anatomical comparisons between the two geometries.

Table of original values for reference	Visible human LES model	Ultra-mill LES model
Total muscle volume (mm^3^)	15,700	1,548
Total cavity volume (mm^3^)	5,400	1,796
Centerline length (mm)	48.3	24.5
Average inner radius (mm)	8.0 ± 1.9	6.4 ± 3.2
Average CM thickness (mm)	2.2 ± 0.6	0.6 ± 0.2
Average LM thickness (mm)	2.2 ± 0.6	0.6 ± 0.2

When the radius was normalized along the centerline of the two models (Figure 3C), there is a noticeable narrowing in the midsection of the Visible Human model, which is not present in the Ultra-mill model. It can be observed that the Visible Human model is much wider at the beginning of the oral end, being more than twice the width of the Ultra-mill model (9.6 vs. 4.0 mm). Distal to 50% along the normalized centerline, the gradients of the two curves began to follow a similar trend, increasing quadratic relationship until the cardia end, by which point the two models seem to share similar radii when measured from their innermost nodes (12.1 vs. 10.1 mm), which suggests that a large part of the differences in cavity volume is due to the differences in length between the two models.

### 3.2 Comparison of intra-luminal pressure development

**FIGURE 4:**
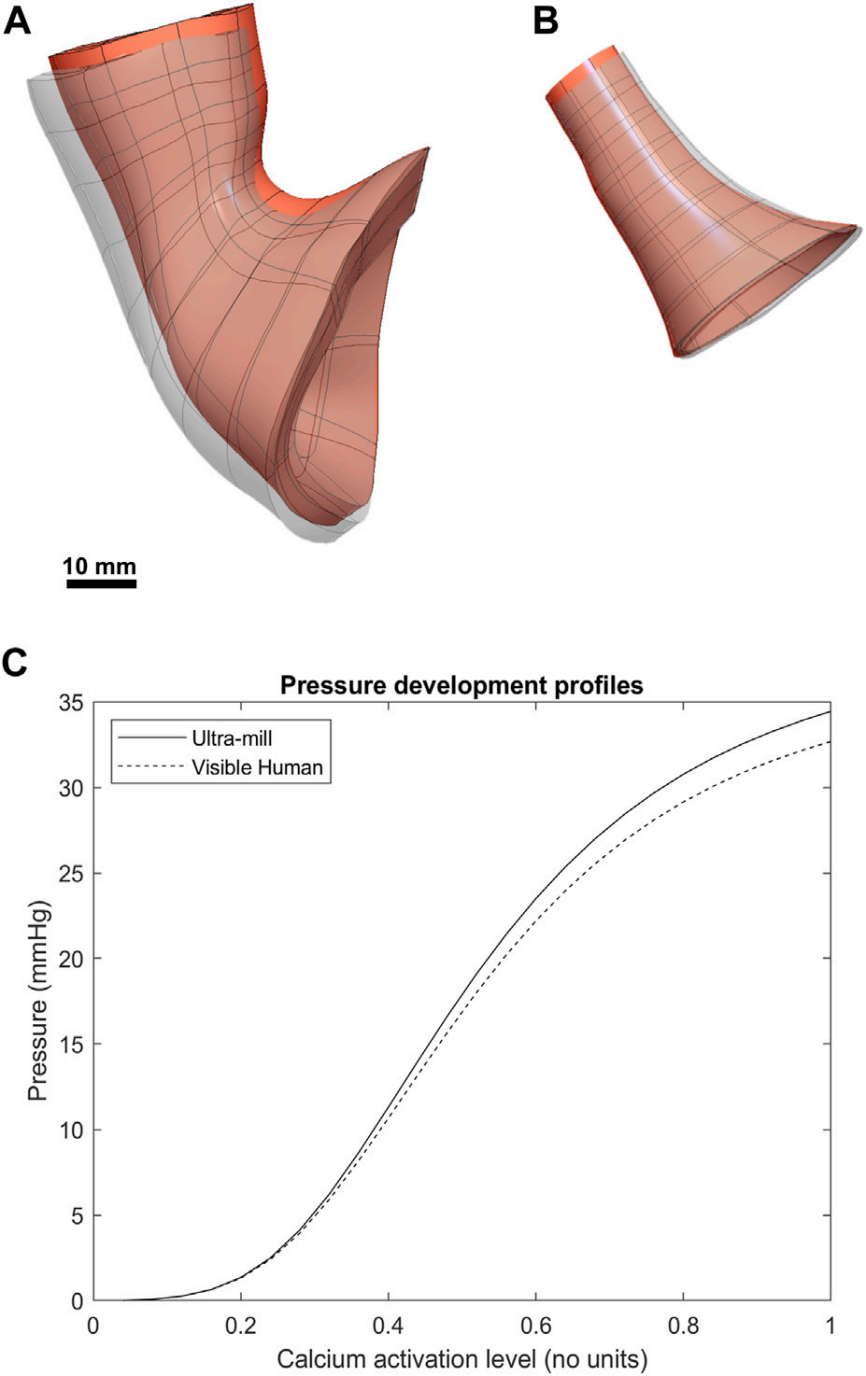
Comparison between the post-contraction behavior of both models. The contracted geometries are visible as greyscale superpositions of the undeformed orange meshes. **(A)** The Visible Human mesh before and after contraction. **(B)** The Ultra-mill meshes before and after contraction. **(C)** The pressure development profiles of the two anatomies over range of muscle activation levels.

Both models underwent post-contraction shortening at the esophageal end (Figures 4A, B), where the upper section post-contraction anatomy was noticeably deformed compared to the undeformed mesh. This effect was more pronounced in the Visible Human model but was also present in the Ultra-mill model. Additionally, the contraction was less uniform in the Ultra-mill model, with lesser contraction along the centerline in the longitudinal direction. There was also an increase in the diameter near the upper esophageal end to compensate for this shortening. Conversely, at the cardia end, the Ultra-mill model shrank radially along the curvature. The shrinkage was present in the equivalent regions of both models despite the difference in their anatomies. The pressure profiles of the two model followed a similar relationship. However, the Visible Human model produced basal resting pressure than the Ultra-mill model (13.8 vs. 14.7 mmHg). The difference between the basal and peak pressures are similarly scaled, Ultra-mill model exhibiting a larger difference (19.8 vs. 18.9 mmHg).

### 3.3 Effect of LES anatomy on intraluminal pressure

**FIGURE 5:**
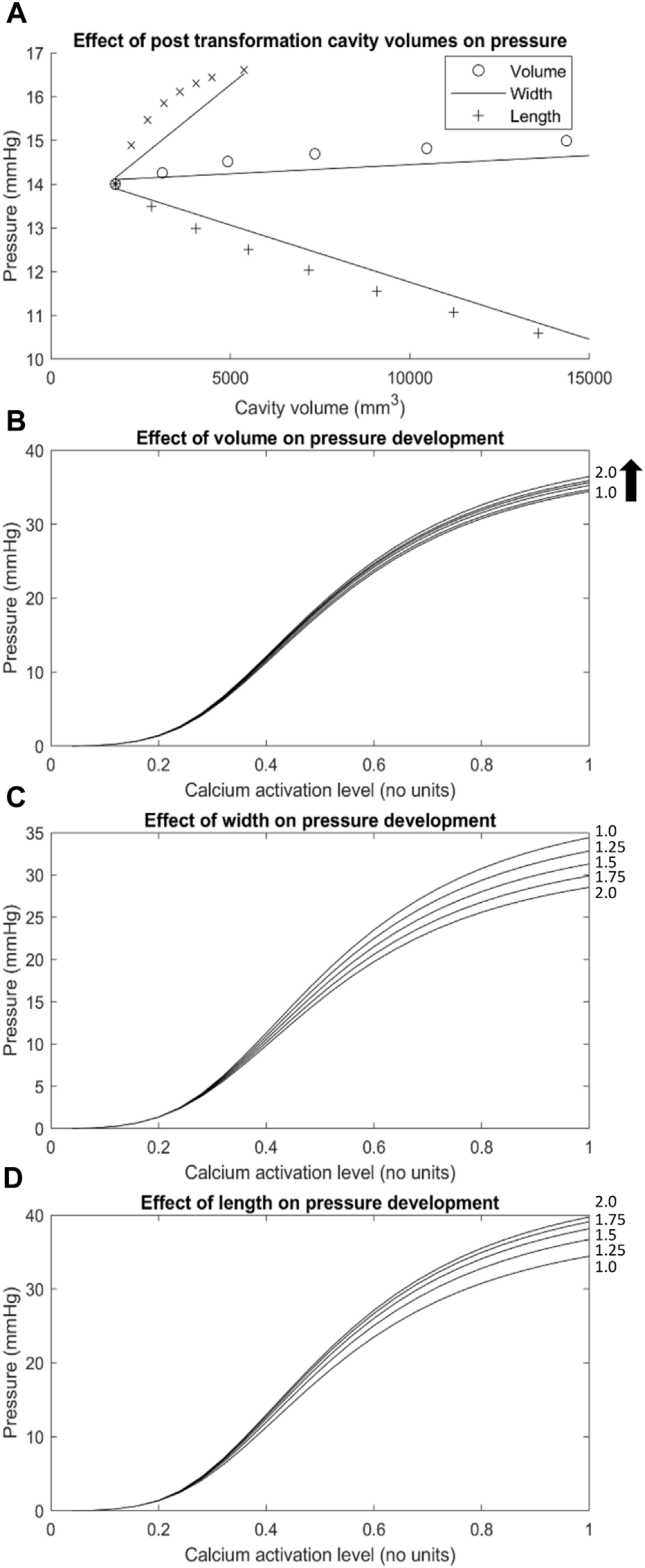
The effect of geometric transformations applied to the Ultra-mill geometry on pressures developed. **(A)** A graph of the basal pressures obtained from the transformed meshes over the cavity volumes associated with their transformations (i.e., The *x* axis of the length trace is the cavity volume of the lengthened mesh, while the *y*-axis is the basal pressure obtained from the transformed mesh). **(B–D)** The changes to pressure development over the range of muscle activation levels tested resulting from geometric transformations including **(C)** width, **(D)** length and a combination of the two defined as **(B)** volume. Geometric transformations are scaled by the factors located on the right-hand side of each trace (i.e., trace 1.25 on graph **(D)** would be a mesh lengthened to 1.25 times the length of the Ultra-mill mesh).

**FIGURE 6:**
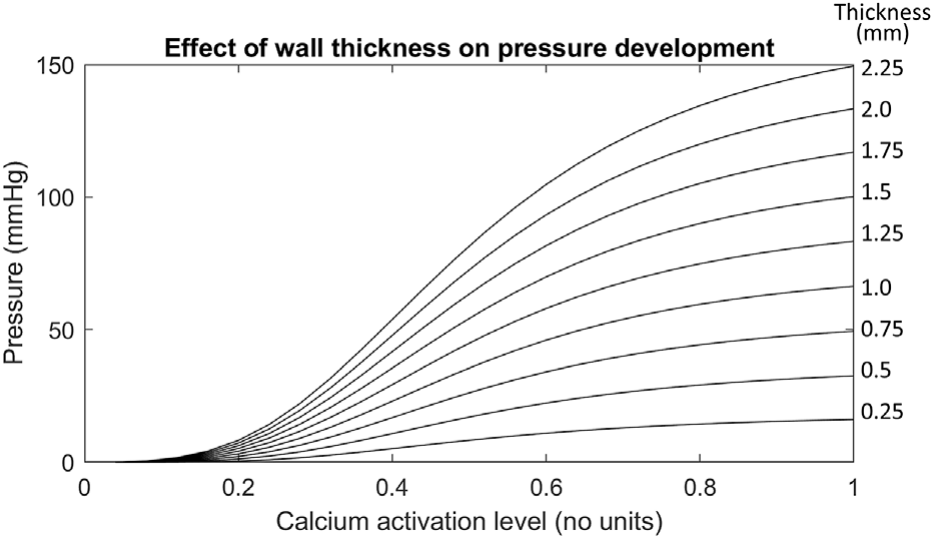
The effect of increasing wall thicknesses on the Ultra-mill geometry pressure development. The wall thicknesses in this test case were made uniform instead of scaling by a factor like in previous tests. The uniform wall thickness is given in mm by the values on the right hand side of each trace.

The volume of the LES had a minor impact on the intraluminal pressure development profile of the Ultra-mill model (Figure 5B). It is important to note that in Figures 5B–D, 6, the basal pressures were offset to 0 in order to allow a clearer comparison between the different transformations. When the volume was increased uniformly by a scaling factor, intraluminal pressures were increased proportionally over the full contraction cycle. By doubling the volume, the intraluminal pressure was increased by 2 mmHg (6%) compared to the baseline at the end of the contraction cycle.

To further differentiate the impact of geometries on intraluminal pressure development, only the length was increased by a scaling factor, and the pressure was proportional to the level of increase over an activation cycle (Figure 5D). By doubling the length, the intraluminal pressure was increased by 5.3 mmHg (15%) compared to the baseline at the end of the contraction cycle. On the other hand, when only the diameter was increased, the intraluminal pressure at the end of the contraction cycle was significantly reduced compared to the baseline (Figure 5C). By doubling the diameter, the intraluminal pressure was decreased by 5.9 mmHg (17%) compared to the baseline at the end of the contraction cycle. It is therefore plausible that the dilation of the lumen of LES had a more significant impact on the intraluminal pressure towards the end of the contraction cycle than the length increase. However, it is also important to note that the increase in cavity volume was not the same between the different transformations (Figure 5A). Relative to uniform dilation of volume, the length had more of an impact on pressure development over a smaller range of volume change than increase in circumference.

Out of all the anatomical considerations, muscle thickness had the most significant impact on amplitude of intraluminal pressure development, as shown in Figure 6. For example, when using a muscle thickness of 2 mm, the intraluminal pressure was increased by 67 mmHg (101%) compared to a 1 mm thickness mesh. Increasing the wall thicknesses also caused large increases to the basal pressure developed, compared to the other transformations. The magnitude of increase amounted to 4 kPa/mm of wall thickness added to both muscle layers.

## 4 Discussion

This study investigated the impact of anatomy of human LES on intraluminal pressure development. To achieve this, a finite element LES model based on a unique ultra-mill dataset was developed and compared against an existing LES model based on the male Visible Human dataset (Yassi et al., 2009). The main finding was that, compared to the Visible Human model, the Ultra-mill model produced a higher intraluminal pressure at every point during an active contraction cycle, likely due to anatomical differences between the two models. Furthermore, perturbation studies of the Ultra-mill LES model anatomy revealed that changes in the length had a more significant impact on intraluminal pressure development than width or volume. Although a one-to-one comparison between muscle thickness and other geometric transformations was not practical as cavity volume did not change, the muscle wall thickness appeared to have the most impact on both basal pressure and the absolute amplitude of intraluminal pressure development.

There were several prominent differences between the shapes of the Ultra-mill and Visible Human anatomies in terms of curvature, length, muscle volume, and radius. These differences could be attributed to a combination of factors, such as, inter-individual variability, preparation method, and state-of-tissue. The most obvious difference was the absence of curvature and cardiac notch in the Ultra-mill model (Figure 3), which could not be simply attributed to the amount of cardia tissue included in the model. The most likely factor was the presence of surrounding tissue. The Ultra-mill specimen was fixed in a vertical direction by pins to a cage during the preparation procedure. Despite the surgeon’s best efforts to mimic its *in vivo* state of the Ultra-mill model, the lack of surrounding cavity structures would have altered its anatomy from its true *in vivo* state.

Another notable factor was the different fixing methods applied, the Ultra-mill specimen was fixed in wax and then milled (Yassi et al., 2010), whereas the Visible Human specimen was cryo-sectioned (Spitzer and Scherzinger, 2006). The two methods required different timeframes to complete and could have had different effects on the specimens. The timing of preparation could be another important factor in the anatomical difference between the two models.

The differences in anatomies between the two models led to difference in intraluminal pressure development. With all the other parameters being equal, the Ultra-mill model produced higher pressures than the Visible Human model (Figure 4), while the profile of development was consistent between the two models. The impact of anatomy was further explored by altering the volume, length and diameter of the Ultra-mill model (Figure 5). By enlarging the cavity volume of the model radially the intraluminal pressure was reduced at the end of the contraction cycle (Figures 5B, C), which is consistent with physical principle. On other hand, lengthening the model led to greater increase actual muscle volume so the intraluminal pressure was higher as the model was lengthened (Figure 5D). These anatomical considerations on intraluminal pressure are especially relevant for the pathophysiology and treatments of hiatal hernia (Fuchs et al., 2021), which includes both anatomical and functional changes that may result in a negative feedback loop that further deteriorates LES functions and increases GERD symptoms.

The importance of individual muscle layer activation was investigated previously (Yassi et al., 2009), so the present study focused on the impact of total muscle thickness on intraluminal pressure development, which had the greatest impact out of all the anatomical considerations (Figure 6). In this case, it was likely that the passive-resistive forces started to become significant due to larger strains being produced. Higher strains from thicker muscular walls may induce greater stresses in the radial direction, increasing stresses applied to the cavity mesh, which could have contributed to the increase in the gradient of pressure to the wall thickness and the observed trend.

There are a number of limitations that warrant further discussion. Although a constitutive law of specifically LES tissue inclusive of shear resistance has yet to be described, experiments could be conducted to estimate the appropriate parameters for the material law employed in the present study (Guccione et al., 1991). More suitable governing equations of electro-mechanical coupling of esophageal smooth muscles could also be adopted (Nicosia and Brasseur, 2002), and while the tissue described by the model was not an exact match, it would be a closer presentation than a cardiac constitutive model. More tissue specific parameter values would also allow better prediction of the biomechanical deformation under various physiological conditions. However, this should be done after a more vigorous analysis of the sensitivity of those parameters, which was missing in the present study due the limited choice of the constitutive law. Another limitation of the current approach is the indeterminacy of the LES region between the datasets used. Without determining the detailed anatomical features of muscle fibers and/or functional measurements, it was difficult to determine the exact position of the LES in both the Ultra-mill and the Visible Human models, and therefore made exact one-to-one comparison between the two models challenging. This was most significant when considering the effects of length and cavity volume on the pressure developed. Length and volume factors were also influenced by the difference in the tissue preparation methods between the data sets. Alternative imaging techniques, such as MRI (Roy et al., 2012), and micro-CT (Vegesna et al., 2013), could provide further functional and detailed micro-structural information of the LES for modeling investigations, and statistical meaningful comparisons between different states. Finally, a larger data set of similar tissue acquisition methods would allow for the validation of the model. A principal component analysis could be performed on the anatomies to determine the defining characteristics of LES anatomies and their relation to pressure. The anatomies could then be grouped by their characteristics, potentially allowing the pressure response of each model to be determined without performing individual biomechanical analysis. Such an approach would significantly improve the clinical applications of the biomechanical model by expediting the process of getting patient-specific analysis.

In conclusion, a subject specific LES model was reconstructed from a unique ultra-mill dataset and compared against a benchmark model. Differences in anatomies and the resultant changes in intra-luminal pressure development were observed. With more tissue-specific material laws and parameters, the models could be refined and applied to inform the pathophysiological impact of LES abnormalities and predict the outcomes of surgical interventions.

## Data Availability

The original contributions presented in the study are included in the article/supplementary material, further inquiries can be directed to the corresponding author.

## Appendix 1

**TABLE A1:** The effect of changing the parameters of the strain energy equation was investigated.

Percentage increase in parameter	Basal pressure Δ%			
	C_1_	C_2_	C_3_	C_4_
25	0.97	1.16	0.00	0.01
50	1.74	1.88	−14.25	0.03
75	2.36	2.26	−19.26	0.04
100	2.89	2.37	−23.40	0.06

Perturbation analysis of the sensitivity of the material parameters in Eq. 2.2. Base values of C_1-4_ are 5, 195, 185 and 0.1 respectively.

## References

[B1] Acharya S. Halder S. Kou W. Kahrilas P. J. Pandolfino J. E. Patankar N. A. (2021). A fully resolved multiphysics model of gastric peristalsis and bolus emptying in the upper gastrointestinal tract. Comput. Biol. Med. 143, 104948. 10.1016/j.compbiomed.2021.104948 35091365 PMC9014465

[B2] Du P. Yassi R. Gregersen H. Windsor J. A. Hunter P. J. (2016). The virtual esophagus: Investigating esophageal functions *in silico* . Ann. N. Y. Acad. Sci. 1380, 19–26. 10.1111/nyas.13089 27310396

[B3] Fox M. R. Bredenoord A. J. (2008). Oesophageal high-resolution manometry: Moving from research into clinical practice. Gut 57, 405–423. 10.1136/gut.2007.127993 17895358

[B4] Fuchs K.-H. Lee A. M. Breithaupt W. Varga G. Babic B. Horgan S. (2021). Pathophysiology of gastroesophageal reflux disease-which factors are important? Transl. Gastroenterol. Hepatol. 6, 53. 10.21037/tgh.2020.02.12 34805575 PMC8573365

[B5] Furlow B. (2004). Barium swallow. Radiol. Technol. 76, 49–58. quiz 59–61. Available at: http://www.ncbi.nlm.nih.gov/pubmed/15503719. 15503719

[B6] Gerneke D. A. Sands G. B. Ganesalingam R. Joshi P. Caldwell B. J. Smaill B. H. (2007). Surface imaging microscopy using an ultramiller for large volume 3D reconstruction of wax- and resin-embedded tissues. Microsc. Res. Tech. 70, 886–894. 10.1002/jemt.20491 17661361

[B7] Goyal R. K. Chaudhury A. (2008). Physiology of normal esophageal motility. J. Clin. Gastroenterol. 42, 610–619. 10.1097/MCG.0b013e31816b444d 18364578 PMC2728598

[B8] Guccione J. M. McCulloch A. D. Waldman L. K. (1991). Passive material properties of intact ventricular myocardium determined from a cylindrical model. J. Biomech. Eng. 113, 42–55. 10.1115/1.2894084 2020175

[B9] Hunter P. J. McCulloch A. D. ter Keurs H. E. (1998). Modelling the mechanical properties of cardiac muscle. Prog. Biophys. Mol. Biol. 69, 289–331. 10.1016/s0079-6107(98)00013-3 9785944

[B10] Krugmann J. Neumann H. Vieth M. Armstrong D. (2013). What is the role of endoscopy and oesophageal biopsies in the management of GERD? Best. Pract. Res. Clin. Gastroenterol. 27, 373–385. 10.1016/j.bpg.2013.06.010 23998976

[B11] Liao D. Zhao J. Yang J. Gregersen H. (2007). The oesophageal zero-stress state and mucosal folding from a GIOME perspective. World J. Gastroenterol. 13, 1347–1351. 10.3748/wjg.v13.i9.1347 17457964 PMC4146917

[B12] Mahadevan V. (2020). Anatomy of the oesophagus. Surgery 38, 677–682. 10.1016/j.mpsur.2020.08.004

[B13] Mitchell D. J. McClure B. G. Tubman T. R. (2001). Simultaneous monitoring of gastric and oesophageal pH reveals limitations of conventional oesophageal pH monitoring in milk fed infants. Arch. Dis. Child. 84, 273–276. 10.1136/adc.84.3.273 11207184 PMC1718697

[B14] Nayar D. S. Khandwala F. Achkar E. Shay S. S. Richter J. E. Falk G. W. (2005). Esophageal manometry: Assessment of interpreter consistency. Clin. Gastroenterol. Hepatol. 3, 218–224. 10.1016/s1542-3565(04)00617-2 15765440

[B15] Nicosia M. A. Brasseur J. G. (2002). A mathematical model for estimating muscle tension *in vivo* during esophageal bolus transport. J. Theor. Biol. 219, 235–255. 10.1006/jtbi.2002.3118 12413878

[B16] Oberhofer K. Lorenzetti S. Mithraratne K. (2019). Host mesh fitting of a generic musculoskeletal model of the lower limbs to subject-specific body surface data: A validation study. Appl. bionics Biomech. 2019, 8381351. 10.1155/2019/8381351 30906423 PMC6398081

[B17] Paoletti G. Melone G. Ferri S. Puggioni F. Baiardini I. Racca F. (2021). Gastroesophageal reflux and asthma: When, how, and why. Curr. Opin. Allergy Clin. Immunol. 21, 52–58. 10.1097/ACI.0000000000000705 33369569

[B18] Roy S. Fox M. R. Curcic J. Schwizer W. Pal A. (2012). The gastro-esophageal reflux barrier: Biophysical analysis on 3D models of anatomy from magnetic resonance imaging. Neurogastroenterol. Motil. 24, 616–625. e269. 10.1111/j.1365-2982.2012.01909.x 22417158

[B19] Savarino E. Bhatia S. Roman S. Sifrim D. Tack J. Thompson S. K. (2022). Achalasia. Nat. Rev. Dis. Prim. 8, 28. 10.1038/s41572-022-00356-8 35513420

[B20] Spitzer V. M. Scherzinger A. L. (2006). Virtual anatomy: An anatomist’s playground. Clin. Anat. 19, 192–203. 10.1002/ca.20330 16565945

[B21] Tutuian R. Vela M. F. Shay S. S. Castell D. O. (2003). Multichannel intraluminal impedance in esophageal function testing and gastroesophageal reflux monitoring. J. Clin. Gastroenterol. 37, 206–215. 10.1097/00004836-200309000-00004 12960718

[B22] Vegesna A. K. Sloan J. A. Singh B. Phillips S. J. Braverman A. S. Barbe M. F. (2013). Characterization of the distal esophagus high-pressure zone with manometry, ultrasound and micro-computed tomography. Neurogastroenterol. Motil. 25, 53–60.e6. 10.1111/nmo.12010 22998376 PMC3530622

[B23] Winans C. S. (1972). Alteration of lower esophageal sphincter characteristics with respiration and proximal esophageal balloon distention. Gastroenterology 62, 380–388. 10.1016/s0016-5085(72)80142-2 5011529

[B24] Wu P. I. Sloan J. A. Kuribayashi S. Gregersen H. (2020). Impedance in the evaluation of the esophagus. Ann. N. Y. Acad. Sci. 1481, 139–153. 10.1111/nyas.14408 32557676

[B25] Yassi R. Cheng L. K. Al-Ali S. Sands G. Gerneke D. LeGrice I. (2010). Three-dimensional high-resolution reconstruction of the human gastro-oesophageal junction. Clin. Anat. 23, 287–296. 10.1002/ca.20941 20169612 PMC4106913

[B26] Yassi R. Cheng L. K. Rajagopal V. Nash M. P. Windsor J. A. Pullan A. J. (2009). Modeling of the mechanical function of the human gastroesophageal junction using an anatomically realistic three-dimensional model. J. Biomech. 42, 1604–1609. 10.1016/j.jbiomech.2009.04.041 19481212 PMC2778051

[B27] Zifan A. Kumar D. Cheng L. K. Mittal R. K. (2017). Three-Dimensional myoarchitecture of the lower esophageal sphincter and esophageal hiatus using optical sectioning microscopy. Sci. Rep. 7, 13188. 10.1038/s41598-017-13342-y 29030643 PMC5640646

